# Challenges and opportunities of remote public involvement and community engagement during a pandemic: refining the MapMe childhood healthy weight intervention

**DOI:** 10.1177/17579139221110015

**Published:** 2022-06-29

**Authors:** L McSweeney, B Arnott, A Jones, G Cain, J Jenkins, A Andras, A Adamson

**Affiliations:** Population Health Sciences Institute, Newcastle University, Framlington Place, Newcastle upon Tyne, UK; Population Health Sciences Institute, Newcastle University, Newcastle upon Tyne, UK; Population Health Sciences Institute, Newcastle University, Newcastle upon Tyne, UK; Public and Involvement Group Parent Representative; Public and Involvement Group Parent Representative; Public and Involvement Group Parent Representative; Population Health Sciences Institute, Newcastle University, Newcastle upon Tyne, UK

**Keywords:** child health, obesity, public health

## Abstract

**Aims:**

Including parents and other stakeholders in the development of interventions to address the sensitive public health issues such as childhood obesity, through public involvement is critical. However, the Covid-19 pandemic has created a challenge for public involvement and engagement activities (PICE). The aim of this paper is to describe the process and challenges of setting up, maintaining, evaluating, and recording impact of three public and stakeholder groups via remote methods in the context of the MapMe2 study during the Covid-19 pandemic. Parental reaction to result letters received as part of the National Child Measurement Programme (NCMP) informing parents of their child’s overweight status is often one of hostility or disbelief. As a result, parents often do not act on these letters to address child overweight. The MapMe2 study is working in collaboration with the NCMP and local authorities, building on previous work (MapMe) and aims to support parents of primary school–aged children to recognise and maintain a healthy weight in their child. The existing MapMe Intervention includes an enhanced NCMP child weight result letter, supplemented with Body Image Scales (BIS), and an intervention website with material to support healthy eating, physical activity, and signposting supporting information. The intervention was to be refined and the evaluation informed with PICE input.

**Methods:**

Covid-19 restrictions meant that planned face-to-face PICE methods had to be altered with all recruitment, all correspondence, and activities taking place remotely. A Parent Involvement Panel (PIP), a child panel, and an expert panel were established. Several adaptations were made to accommodate a new way of involving the public in research.

**Results/Conclusions:**

Working remotely created many challenges and was a learning experience for all involved. However, an active group was successfully established. Using continuous assessment and evaluation methods, we were able to demonstrate successful involvement and engagement in the refinement of the MapMe2 study. Through the sharing of PICE methods practice, this paper adds to the literature, the value of partnership working.

## Background

Childhood obesity (OB) is both a national and international public health priority.^1–3^ Data from the National Child Measurement Programme (NCMP; a national mandated programme led by Public Health England (PHE)) shows that in 2020–2021, 27.7% of children in England enter primary school at the age of 4–5 years with either overweight (OW) or OB with prevalence increasing to 40.9% in those children aged 10–11 years, in their final year of primary education.^4^ The prevalence of OB has been found more than twice as high in the most deprived areas compared to the least deprived areas.^5^ An increase in the deprivation gap for OW/OB has been observed between 2006–2007, when annual monitoring began, and most recent measures in 2020–2021; this disparity was particularly apparent for those children in the older age group. The prevalence of childhood OW/OB and the evidence of widening inequalities are alarming; having OW/OB during childhood can adversely impact both short- and long-term physical and psychosocial outcomes since excess weight is known to track across the life course.^1,6,7^

In England, the NCMP reports the weight status of 4- to 5- and 10- to 11-year-olds to parents via letter.^8^ Parents do not always recognise OW in their child^9^ and are often surprised by and mistrust the result.^10,11^ They perceive advice given in the NCMP letter to seek medical help from a GP for their child’s OW as inappropriate, and may not take action.^12^ Receiving OW feedback can be dismissed by parents or perceived as an issue for other families.^10^ Given that the NCMP will continue as a monitoring device^12^ and that results will continue to be fed back to parents, it is essential that appropriate interventions are developed to enhance and supplement the letter to support families to take action to maintain a healthy weight in their child. Involvement and engagement activities are critical to the development and evaluation of interventions. For the purposes of this article, we discuss public involvement and stakeholder engagement, the former referring to lay individuals and the latter to practice partners and/or public health professionals. These activities are summarised under the term Public Involvement and Community Engagement (PICE).

In the UK, the National Institute for Health and Care Research (NIHR) highlights active PICE as an important component of research studies and state that patient and public involvement (PPI; part of PICE activity) ‘can improve the quality and relevance of research, as well as serving the broader democratic principles of citizenship, accountability and transparency’.^13^ Involvement is an active partnership between patients, carers, and members of the public with researchers that influences and shapes research.^13^ INVOLVE, a UK-based public participation charity,^14^ condensed Arnstein’s ladder of participation^15^ into three steps: ‘consultation’, ‘collaboration’, and ‘lay control’.^16^ Public PICE contributors can be conceptualised into several types,^17^ including:

- The expert in lived experience (through their lived experience of a condition or situation, PICE contributors are able to consider the acceptability and feasibility of research methods);
- The bridger (bridges the communication gap between researchers and the public making research more relevant and accessible);
- The motivator (PICE contributors increase researchers’ motivation/enthusiasm by emphasising how the research will benefit people);
- The passive presence (PPI contributors can change the way that professionals think just by being present at meetings).

PICE is an activity that young people and children can also contribute to, especially if the research will directly impact them. Children can contribute as researchers or as members of an advisory panel member.^18^ Children may see things differently and ask questions that an adult has not considered.^19^ Involving children in research can provide many benefits, such as, improving the suitability of research tools for use with other children. Taking part in research may increase children’s self-confidence, self-esteem, and problem-solving skills.^20^

While it is increasingly accepted that PICE is an essential aspect of research with numerous benefits, a lack of understanding, support, funding, and time may impact the researchers’ motivation and ability to meaningfully incorporate PICE activities.^21,22^ Some researchers report apprehension in involving the public and stakeholders, due to uncertainty of new ways of working^21^ and increased workloads.^22^ Careful planning, training, a clear definition of roles, and adequate funding may improve the success of PICE.^23^ It is also important to evaluate and demonstrate the impact PICE has on the research. The research team roles, process of PICE implementation, and research teams’ values of PICE should also be appraised and reported.^23^ These reports can be used to share best practices wider within the research community^24^ and inform of the complexities of evaluating PICE.^23^ A key limitation of the PICE evidence base is described as the poor quality of reporting impact.^25^

Evaluating and recording impact also aids provision of feedback to PICE contributors, which they report being an important aspect of involvement. Children too request that feedback of the impact of their involvement is provided, to show their involvement is worthwhile.^26^ ‘Simple feedback between PICE contributors and researchers can improve the involvement process, spur mutual learning, and change researchers’ mindsets and future practice’.^27^

Restrictions during the Covid-19 pandemic have greatly increased the challenges in involving and engaging the public and stakeholders in research. When conducting remote PICE through digital meetings, there is a need to be aware that digital communication, such as the use of video platforms, poses a different set of challenges than in-person communication. Additional efforts are required from researchers, to reach the same level of input, information sharing, and collaboration.^28^ However, given the high prevalence of childhood OW and OB with prediction this will have increased following the Covid-19 pandemic,^4,29^ parental, child, and stakeholder input and action is essential and cannot be postponed at this point in time.

The aim of this paper is to describe the setting up, maintenance, evaluation and recording impact of involvement with three remote PICE groups for the MapMe2 study during the Covid-19 pandemic. These comprised a Parent Involvement Panel (PIP), a child involvement panel, and an ‘expert’ stakeholder engagement group. The paper describes individual group recruitment, how communication and engagement was initiated and maintained, how challenges were resolved, the level of involvement, how data were gathered and utilised from each group, and the impact on the MapMe intervention development. The MapMe2 study methods are briefly described; detail will be published separately.

## Methods

### Refinement of existing intervention

The original ‘MapMe’ intervention developed in previous work^30^ includes Body Image Scales (BIS) of known weight status, showing images of underweight to very OW children of NCMP age, to help parents recognise child weight status. In addition, the intervention included information on healthy eating, physical activity, consequences of child OW, and further support, and was developed in paper- and web-based formats. MapMe was tested in a preliminary study with ~300 OW/OB children. Children whose parents had access to MapMe showed improved body mass index (BMI) *Z* scores after 1 year.^31^ A definitive trial, working with the NCMP and nine local authorities (LAs), is now underway to confirm these findings in a larger study: The MapMe2 study is funded by the NIHR (https://fundingawards.nihr.ac.uk/award/NIHR127745). As part of this definitive trial, the plan was to refine and update the intervention, and to evaluate its effectiveness and cost-effectiveness. To understand how the intervention works and to inform future implementation, a sub-study was also being conducted.

### Incorporating PICE into the MapMe2 study

All PICE activities were co-ordinated by a PICE co-ordinator and a research associate assigned to work solely on the day-to-day running of the study PICE activities, analysis of feedback, reporting of results, and dissemination. A budget for the three PICE groups including remuneration purposes and training was included in the study costs.

### PICE recruitment

PIP: comprising parents/carers of primary school–aged children who were recruited through social media, University staff webpages, ethnic minority groups, a group for parents who had a child with OW, and through known contacts.

A child involvement panel: 10- to 11-year-olds were recruited; as part of the MapMe2 sub-study, 10- to 11-year-olds will independently complete questionnaires and dietary intake diaries. Children were recruited through known staff contacts, a necessarily pragmatic decision, because at the time, schools and children’s groups were closed due to Covid-19.

An ‘expert’ stakeholder panel: Public health practitioners, academics, school nurses, and LA/government stakeholders identified through known contacts, public health colleagues, and practice partners were invited to form an ‘expert’ panel.

### Recognition of involvement

To acknowledge PICE members’ input, using the NIHR Payment guidance for researchers and professionals,^32^ the PIP were provided alternative ways in which to be remunerated; these included shopping vouchers, making a donation to charity, a certificate of achievement, a reference for a job/college application, and opportunities to take part in PPI training. Children were offered online shopping vouchers for their time.

### Communication methods and materials

The pandemic meant that traditional PICE methods, such as face-to-face meetings and focus groups, were not possible; therefore, all correspondence and meetings took place remotely.

PIP: As parents are actively involved in all aspects of the project throughout and not just the refinement stage, they were consulted on how best to be involved. Methods suggested included email, Zoom meetings, WhatsApp, text, and telephone. A mobile phone was purchased for the research team to facilitate requests. To allow a range of information and communication methods to be accessed, a PIP ‘Welcome and Training Pack’ was developed in both digital and paper formats. Furthermore, a series of short, animated training videos and research team–presented study information videos were developed and shared.

Child panel: The child panel was involved on two occasions and communication was through their parents, with contact made by email.

Expert panel: Panel members were consulted several times during the study and communicated with researchers and other panel members via email and Zoom.

### PICE activities

The PIP was involved directly throughout the research cycle, providing input into the direction of the study, refining methods of data collection, contributing to funder reports, informing refinement and evaluation of the intervention, and dissemination. Representatives also attended the Trial Steering Committee meetings (remotely). Regular newsletters with study updates and information of how PIP input shaped the study development were distributed quarterly to PIP members. The Child and Expert panel were consulted periodically, when required, to advise on certain study aspects, such as, the questionnaires to be completed by children in the sub-study (child panel) and the NCMP enhanced result letters (expert panel).

Meetings by Zoom, attended by adults only, were video and audio recorded (with permission) to assist researchers with meeting recall and deleted after the transcriptions were downloaded. Transcriptions were anonymised, as was feedback and comments received by email. Commonalties and divergences from feedback and discussions were identified by the PICE researcher and coordinator.

### PICE activities evaluation

Continuous evaluation of activities to facilitate understanding of the impact of the PICE activities and to recognise what worked and what could be improved was implemented using the School for Primary Care Research record of involvement and engagement activities template.^24^ The template helps to detail PICE activities, outlines who was involved, what actions were taken, the impact of the involvement, and how challenges were dealt with.

Also utilised was the Public Involvement Impact Assessment Framework (PiiAF)^33^ as recommended by NIHR^24^; this enables researchers to think about values, approaches, research focus, and practical issues which may impact PICE activities.

To evaluate the groups’ involvement at an individual level, the PIP and child panel were invited to complete a survey about their involvement. To ascertain views of the expert stakeholder group and the research team, they were invited to participate in an involvement values task^33^ using the interactive platform Padlet.^34^

## Pice Evaluation Results

### Planning and assessment of PICE

The process of planning for PICE, the challenges faced, and how impact would be identified using PiiAF^33^ is given in Supplemental Appendix 1. The main component impacting PICE for the MapMe2 study was the global Covid-19 pandemic which affected recruitment and communication methods. The research team were mindful that during the UK lockdowns, when schools were closed, some parents were working from home, home-schooling children and coping with the impacts of Covid-19. The research team tried to ensure the PIP group was ethnically diverse and that inequalities in participation by digital access were addressed by using a variety of remote methods.

### PICE membership

PIP: Following the advertisement of the involvement opportunity in June 2020, we recruited 21 members; this included 19 females and 2 males, 2 members were known to be from ethnic minority backgrounds. Successful recruitment was mainly achieved through social media (Facebook) posts and Newcastle University staff webpages. Of those recruited, 11 members responded to one request for task participation/feedback, with 7 members responding/contributing on more than one occasion. In January 2021, we contacted PIP members who were not responding to determine if they still wanted to be involved. Members were asked to opt-in if they wished to remain; eight members requested to remain. We retained the two male parents/carers, but the ethnic diversity decreased. We advertised for more members throughout, with particular focus on links/contacts to increase our membership diversity and to include parents who had received an NCMP result letter stating their child was OW/VOW. Between January and August 2021, we recruited two more female members, one of whom had received an OW NCMP letter.

Child panel: We involved six 9-year-old children to help/advise with tasks.

Expert panel: 13 expert members contributed to the study refinement on four occasions. Supplemental Appendix 2 illustrates the numbers and sex mix of each group and the professional roles of the expert panel.

### Level of PICE involvement

Most involvement for the MapMe2 study was in the form of ‘consultation’, that is, seeking members’ views to inform decision making. However, ‘collaboration’ (an active ongoing partnership between PICE and research team members) was also apparent with several PIP members remaining active throughout and co-writing/contributing to the study update report and this publication. Members contributed their lived-in experiences, which was crucial for the development of the MapMe2 study and materials. Furthermore, as described by Oliver et al.,^16^ the PICE groups could be described as also contributing to the study in the roles of ‘bridger’, ‘motivator’, and as a ‘passive presence’^16^ (Supplemental Appendix 3). The PIP had mixed methods of involvement, whereas the child panel’s level of involvement was consultation only, and the expert panel involvement was both, consultation and collaboration.

### PICE and record of impact

As demonstrated in Supplemental Appendix 4, PICE contributed to study team decisions and final study methods in substantial ways. The PIP contributed to the study throughout, with an average of 2–3 members involved in each task, mostly by email. The child panel were involved at two time points, June 2020 (*n* = 5) and March 2021 (*n* = 4), by email. The 13 expert panel members contributed on four occasions, 9 December 2020 (*n* = 5) 17 December 2020 (*n* = 3), both meetings using the Zoom online platform. Feedback by email was received in January 2021 (*n* = 3) and June 2021 (*n* = 3).

All groups were asked to provide feedback of their PICE experience and involvement with the study. Seven parents responded – in the main, parents were happy with the communication methods and the amount of information shared by the team, only one parent felt too much information was provided. The tasks were reported as being easy to understand (6/7) with one parent commenting on how much they enjoyed being part of the study.

Four children completed the online survey, they all stated being happy to help with the research and found involvement interesting. They also stated that researchers should contact schools or use social media to encourage more young people to be involved in research.

The research team/expert panel PICE evaluation feedback was limited. However, those that were able to contribute rated study PICE highly. The importance of involving parents in the development of the study was deemed essential. Also highlighted was the need of public and stakeholders to feel the research was being conducted ethically, which would then resonate its findings/outcomes with the parents/families for whom the research was about.

Supplemental Appendix 4 summarises the three groups’ involvement, which tasks they contributed to, the numbers involved, the timeline of the involvement/contribution, challenges encountered, action taken, impact of involvement, and method of feedback to PICE group. The challenges of remote working predictably included issues with Internet connections and changes to recruitment methods. However, the need for more clarity in describing instructions for certain tasks, which would have been easier to do face-to-face, was made apparent.

The main impact findings were:

1. The study materials (questionnaires, Body Image Scales etc.) were revised, in light of involvement, to be more appropriate, acceptable and user-friendly.
2. Communication methods, following PICE feedback, were revised to be more accessible and to enhance remote working.
3. The intervention (NCMP letter and intervention website) was revised following involvement, to be more acceptable and clearer.
4. Study governance (Steering Group committee) revised to ensure remote involvement was accessible.
5. Dissemination – methods were adapted to ensure accessibility.

## Discussion

This paper describes the process and challenges of setting up, maintaining, evaluating, and recording the impact of PICE in the MapMe2 study during the Covid-19 pandemic. Effective PICE was achieved using remote methods, although some methods needed to be adapted; a high level of involvement, as demonstrated in our study, was possible.

Public and stakeholder input for the MapMe2 study was crucial for intervention development, refinement, and planning for evaluation; intervention effectiveness is strongest when people with lived experiences are involved as research partners.^27^ Parental involvement in health research not only ensures the research is more relevant and meaningful but is also empowering and may increase awareness of health issues and the likelihood of making changes in the area of focus,^36^ in this case, maintenance of a healthy weight in their child.

Despite concerns about having to rely solely on remote methods due to the pandemic, we acknowledged the importance of adaptation. We created online and paper welcome/information packs, recording study/training information videos, and provided alternative communication methods. While using remote methods generated many challenges, including learning ‘Zoom culture’, reliance on good Wi-Fi networks,^37^ and risk of reducing diversity of participation by parents, such as those from low-income backgrounds,^38^ there were some positives of remote contact/communication. Parents could contribute from any location in their own time^37^ without having to travel,^39^ which for those juggling home-working and child care was beneficial. Also, as the pandemic progressed and people became more accustomed to using online platforms, they may have felt more comfortable being able to contribute from home.^37^ Notwithstanding these additional challenges, we recruited and maintained a core number of parents in the PIP which we attribute to regular communications with the PIP group. Parents were contacted at regular intervals to assist/work on study tasks, while being mindful of not overly burdening; we sent task reminders (parental request). Feedback was sent to PIP members quarterly to inform of their contributions and outcomes of their contributions. This, we trusted, helped parents feel part of the team and involved in study progress despite not meeting in person. We understood this to be one of the most important aspects of PICE for contributing members.^17,26^ Although the child panel was consulted on only two occasions, four of the children completed the involvement feedback survey and responded positively to being involved.

For the research team and expert panel too, the benefits of parental and stakeholder involvement are numerous; PICE can help identify issues and details that researchers may not have been aware of^36^; for example, in this study, context and use of language concerned with child OW and ways in which sensitive information should be presented to parents/families. Also, an increased pool of expertise and opinions leading to greater rigour in decision making and overall quality of results which may increase credibility of the research with other professionals.^36^ We provided the research team and expert group an opportunity to participate in the values based online exercise based on the PiiAF^33^; however, participation was low. Reasons for this are likely to be due to lack of time and not having opportunities to meet face-to-face as opposed to not valuing PICE. It was apparent that PICE was valued in the MapMe2 study being fully funded and including dedicated staff resource.

It should be noted that ongoing PICE throughout a study is challenging and takes time, resources, and energy. This study was fortunate in that, adequate funding was costed for PICE with allocated team members responsible for the implementation, and a payment policy and remuneration funds for contributors. This is contrast to the past when PICE was perhaps often at risk of being a ‘tick-box’ exercise and reflects that the value of public and stakeholder recognition is increasingly being acknowledged.

However, although PICE recognition is growing, evaluation and reporting of impact is still lacking^25^ with no standard method for capturing and reporting impact.^40^ We were mindful that continuous monitoring and evaluation would allow us to systematically record the data/feedback received and observe how PICE contributions were impacting development of the MapMe2 study. Such information is important for reports and feedback to funders; however, the NIHR highlights the need for tools that will not only collect feedback and capture impact of involvement, but will also share learning, which is focused on improving, rather than just justifying the value of partnership (PICE) working.^40^

## Strengths and Limitations

Several strengths can be highlighted. Two research team members were funded to focus on PICE. Different perspectives were well represented by three different groups, that is, parents, children, and professional stakeholders. The research team were mindful of the quality of reporting impact and planned for this accordingly. We were able to maintain regular communication with PICE members by providing alternative methods and provided feedback on a regular basis.

Limitations include the following: lack of face-to-face meetings may have impacted the level of involvement from the PICE groups. Being able to establish a group rapport with PICE and research members may have encouraged a greater level of confidence and involvement than was achieved. There was a lack of formal collection of PICE members’ ethnic background which would help to ensure transparency and promote future reproducibility.^38^ We were unable to access child panel members through usual channels, and the pragmatic approach used might mean these children were from better-educated families and so not a representative sample. Finally, having to adapt quickly to using remote methods, we may have unintentionally excluded parents from a wider sample due to digital poverty/exclusion.

## What we Learned about Remote Pice Activities

- Have a named PICE person/contact;
- Ensure adequate time and resources are allocated;
- Provide alternative methods of communication/feedback;
- Ask contributors how they would like to be remunerated for their time;
- Make sure task instructions are clear; you may need to provide more guidance using remote methods;
- Encourage PICE contributors to seek help/ask questions if they are unsure;
- Provide regular feedback; let members know what they have achieved and the impact they have had on the study;
- Have several methods for researchers/professionals to provide evaluation feedback.

## Future Work

The MapMe2 study commenced the trial with nine LAs, schools, families, and the NCMP in November 2021. At the time of writing, the PICE groups continue to be part of the process, working remotely, and will be involved in data analysis, intervention monitoring, and dissemination activities.

## Conclusion

Despite the challenges issued by the Covid-19 pandemic, we successfully established and engaged with three PICE groups. By taking on board the feedback from our PICE panels, adapting to remote methods, and by using appropriate evaluation and recording of impact methods, we are able to demonstrate successful involvement and engagement in the refinement of the MapMe2 study. We have committed considerable time and resources to achieve this remotely, but we are assured that PICE is thoroughly embedded within the project and having a positive impact.

## Supplemental Material

sj-docx-1-rsh-10.1177_17579139221110015 – Supplemental material for Challenges and opportunities of remote public involvement and community engagement during a pandemic: refining the MapMe childhood healthy weight intervention Click here for additional data file. Supplemental material, sj-docx-1-rsh-10.1177_17579139221110015 for Challenges and opportunities of remote public involvement and community engagement during a pandemic: refining the MapMe childhood healthy weight intervention by L McSweeney, B Arnott, A Jones, G Cain, J Jenkins, A Andras and A Adamson in Perspectives in Public Health

## Notes

i. Note, 2020–2021 figures are based on a weighted sample due to a smaller sample of measurements being collected in comparison to previous years due to the Covid-19 pandemic.
