# Supplementary material for: Challenges and opportunities of remote public involvement and community engagement during a pandemic: refining the MapMe childhood healthy weight intervention
Source: Perspect Public Health. 2022 Jun 29;142(4):224–30. doi: 10.1177/17579139221110015 (PMC9284084; doi:10.1177/17579139221110015)
Supplement: sj-docx-1-rsh-10.1177_17579139221110015 – Supplemental material for Challenges and opportunities of remote public involvement and community engagement during a pandemic: refining the MapMe childhood healthy weight intervention [file sj-docx-1-rsh-10.1177_17579139221110015.docx]

**Appendices**

Appendix 1: Table 1: Assessment of study PICE planning and reflection using PiiAF [25]

|  | **Planning** | **Reflection** |
| --- | --- | --- |
| **Values** | PICE activity within the project is an essential part of the research process and a requirement of the funders. The host research site, Newcastle University recognises the importance of PICE contribution, and the Faculty of Medical Sciences has a dedicated Involvement and Engagement team to provide training and advice to researchers. A study research associate has been assigned to work solely with the PICE members and to oversee the PICE activities. All research team members appreciate the importance of PICE and the benefits its involvement brings to the research process in the development and shaping of the study. PICE activity and updates will be included in the monthly team meeting agenda and team members and professional colleagues (stakeholders) will be asked to participate in a values evaluation exercise. |  |
| **Approaches to PICE** | A parent involvement panel will be set up comprising parents/carers of primary school aged children. This will ensure that we are consulting with and including the users that the study is designed for. Two parents will be invited to sit on the Trial steering group committee to ensure parent representation in the study oversight. We also recognise the importance in involving all stakeholders in the research process. We are fortunate in that we can consult with an expert panel, comprising academics, psychologists, public health partners and school nurses in matters relating to clinical and public health aspects of the study. Furthermore, we will consult with a panel of 10-11-year olds to ensure tasks intended for the children taking part in the study are relevant and acceptable. |  |
| **Research Focus and study design** | The research is the intervention/development and evaluation of a child weight maintenance tool. Working with the National Child Measurement Programme (NCMP), when parents receive their child’s measurement result, they will be directed to a website where they can view 3D images of their child’s weight status and receive support and information for maintaining a healthy weight in their child.  This study builds on previous work and several aspects of the intervention require updating and/or refinement. This includes the body image scales, website and content and the NCMP result letter. The intention is to have involvement at all stages of the research from the development of the materials, refinement of methods and process evaluation of a sub-study to measure impact. Methods to involve/engage include meetings, focus groups, written feedback and involvement in the Trial Steering Committee. | Challenges initially were to do with ability to recruit a diverse range of parents/carers and to keep them engaged throughout the study. However, Covid-19 struck just as the study was beginning, this meant that normal face-to-face methods had to be adapted and all communication methods placed online using remote methods. |
| **Practical issues** |  | The main practical issues that impacted PICE was Covid-19 and the need to change our ways of thinking and engaging. We consulted with the parent group on this issue and determined preferred methods of communication and sharing of materials and feeding back views. We also had to learn how to use Zoom for meetings and communication. We were conscious that during lock-down, many parents were home-schooling their children whilst working from home. Therefore, we were conscious of maintaining the balance of requests for help, keeping interest/engagement and not over-burdening parents with too many tasks.  We also identified that as we were not able to meet face-to-face, we were unable to develop a group rapport that we would have overwise been able to do so. This meant we were not able to engage in informal conversations and gauge levels of interest/need for extra support and/or training. Thus, we decided to develop a parent involvement panel Welcome pack and information manual that could be posted as a hard copy or shared online. We also produced some videos with key staff members explaining different aspects of the study to make the involvement more personable. Animated training videos were also developed and shared to enhance involvement and offer support for further learning if wanted.  We were aware that the diversity of the group was limited, and we made every effort to contact groups/ partners that may be able to help. We were aware also, that as all communication was now taking place online, this may have reduced the diversity of those able to participate further. However, positives were that it did not limit the geographical reach of parents and they were able to participate and contribute when it was suitable for them. |
| **Identifying the impacts of PICE in research** | How?  Continual analysis of PICE input and the impact it has on the research development and refinement.  PIP member testimonies/survey of their involvement.  Surveying child panel of their involvement.  Ask research team members/stakeholders to identify impact of user involvement | **Researchers**: PICE in this project allowed the research team to learn more about PICE and the benefits and challenges it brings. This knowledge undoubtedly will enhance transferable skills for future projects/work with the public.  **PICE members**: Involvement allowed increased knowledge and skills (testimonies from PIP members).  **Research**: Better quality and relevance of research design and delivery/ data collection and dissemination to have a greater impact.  . |

Appendix 2: Table 2: PICE group numbers, gender, and professional role of expert panel

| **PICE Group** | **No. in group/gender** | **Ethnic diversity group representation** | **Professional role (expert panel only)** |
| --- | --- | --- | --- |
| **Parent panel** | 2020: n= 21 (19 F/ 2M)  2021: n= 10 (8F/ 2M) | 2020: n= 2  2021: n= 0 | N/A |
| **Child panel** | n= 9 (all F) | Not known | N/A |
| **Expert panel** | n= 13 (6F/ 3M) | n=1 (known) | - Public health academics n=3 - Public Health England professional n=1 - Local Authority representatives (NCMP team) n=2 - Clinical psychologist n=2 - Public Health Practitioner n=2 - School Nurse n=3 |

Appendix 3: Table 3: PICE contribution study contributions and level of involvement as described by Crocker et al, (2017) and Oliver et al, (2008)

| **Group** | **Study element** | **Level of involvement** |
| --- | --- | --- |
| Parent Involvement Panel | Enhanced NCMP parent result letter (for MapMe2 study) | Consultation  Lived in experience  Bridger |
|  | Health economics evaluation questionnaires | Consultation  Lived in experience  Bridger |
|  | Health economics mapping activity | Consultation  Lived in experience  Bridger |
|  | Sub-study materials including:   - Child health questionnaires - Study information and consent form - Parent interview topic guide | Consultation  Collaboration  Lived in experience  Bridger |
|  | The intervention website including:   - Body image scales - Website content and format - Functionality/ ease of use of website - Additional website resources (animation of National Child Measurement Process and videos of health practitioner talking to parent and parents talking to child | Consultation  Collaboration  Lived in experience  Bridger  Motivator |
|  | PICE blog post | Consultation  Bridger  Motivator |
|  | NIHR funder report (PICE section) | Collaborator  Bridger |
|  | Representation on Trial Steering committee | Consultation  Lived in experience  Bridger  Motivator  Passive presence |
| Child Panel | Child health economics questionnaires | Consultation |
|  | Sub-study method instructions | Consultation |
|  | Sub-study child health questionnaires | Consultation |
| Expert Panel | The NCMP (enhanced) result letter | Consultation  Collaboration |
|  | Body image scales | Consultation |
|  | Sub-study NCMP staff interview topic guide and questionnaire | Consultation  Collaboration |

Appendix 4: PICE contribution to study using the School for Primary Care Research record of involvement and engagement activities template

| **Type of Activity** | **Details** | **Dates of involvement** | **PICE group (n)** | **Method of communication** | **Challenges** | **Action** | **Impact** | **Feedback** |
| --- | --- | --- | --- | --- | --- | --- | --- | --- |
| **Health Economics Analysis** | Child completed questionnaires | 25^th^ June 2020 | Child panel (5) | Email | Due to the UK lockdown and people working from home, we were unable to recruit children through usual methods and had to rely on known contact’s children to take part | Clarification received that 11yr olds could independently complete the questionnaires | Questionnaires are appropriate for children | Information sheet |
|  | Parent questionnaires | 15^th^ July  13^th^ July – 10^th^ August 2020  16^th^ – 28^th^ September | PIP (2)  PIP (6)  PIP (7) | Zoom  Email  Email |  | Added ‘A&E’ and ‘Doctor’ to clarify meaning of ‘Accident and Emergency’ and ‘GP’ | Questionnaires are appropriate for parents | Quarterly PIP newsbrief |
|  | Discrete Choice Experiment | 14^th^ October 2020  2^nd^ November 2020  29^th^ November 2020  18^th^ November 2020 | PIP (3)  PIP (1)  PIP (1)  PIP (1) | Email  Zoom  Zoom  Zoom | Initial feedback to this task was not as expected. A couple of parents stated they were unsure how to answer. Extra support and instructions were given by email and Zoom | Instructions for completion were made clearer | Task is fit for purpose | Quarterly PIP newsbrief |
|  | Revised instructions | 14^th^ December 2020 | PIP (2) | Email |  | Clarification that instructions were now clear |  |  |
| **Body Image Scales** | Web images | 2^nd^ July 2020  9^th^ July 2020  17^th^ – 26^th^ August 2020  7^th^ January 2021 | PIP (3)  PIP (2)  PIP (3)  PIP (5) | Zoom  Email  Email  Email | One parent had internet connection difficulties. Another parent found it difficult to join Zoom meetings due to home schooling. Parents were encouraged to feedback using other methods | Images were modified to all being in sport’s wear as opposed to underwear.  The faces of the 4-5 yr old boys were modified to look less ‘adult’.  A note to be added with the images to explain why only Caucasian children are represented. | Images are acceptable and relevant for parents.  Caucasian only representation caveat provides acknowledgment of tool limitations | Quarterly PIP newsbrief |
|  | Paper-based images | 29^th^ January 2021  3^rd^ February 2021 | Expert panel (school nurses) (2)  PIP (5) | Email  Email |  |  |  |  |
|  | Text for images | 26^th^ July 2021 | PIP (3) | Email |  | Text for images was added at the bottom of the page | Document more user-friendly | Quarterly PIP newsbrief |
| **PIP** **Preferred Communication Methods survey** |  | 17^th^ July – 23^rd^ October 2020 | PIP (15) | Online survey | Responses to the survey were slow. Holiday time was thought to be a factor, so survey was left open for longer | A study mobile phone was purchased to assist with the requested use of online methods such as text and WhatsApp. | Increased accessibility to PPI participation | Quarterly PIP newsbrief |
| **National Child Measurement Programme – Parent Result Letter** |  | 26^th^ November 2020  3^rd^ December 2020  9^th^ December 2020  11^th^ December 2020  17^th^ December 2020  17^th^ December 2020 | PIP (2)  PIP (1)  Expert Panel (4)  PIP (3)  PIP (1)  Expert Panel (3) | Email  Zoom  Zoom  Zoom  Email  Zoom | Experts expressed preference to discuss the NCMP letters by Zoom, however getting busy experts together at the same time on Zoom was challenging | Singular references to child’s lifestyle removed. Recommendation for 60 mins daily PA should be for all children.  Focus on growth and health rather than weight and shape. The association of weight and health lessened. Subtle phrase changes made. | Letter may be more acceptable to parents | Quarterly PIP newsbrief  Presentation to CI meeting |
| **Sub-Study Materials** | Instructions and questionnaires | 29^th^ March 2021  22^nd^ April 2021 | Child Panel (3)  PIP (3) | Email  Zoom | Feedback from one parent made us realise that feedback task instructions needed to be made more explicit as there was some confusion over the task | Questionnaires made more mobile ‘friendly’.  Numbering on questions removed.  Estimated time needed for questionnaires added.  Information that questionnaires can be completed in stages added.  Child-completed dietary intake questionnaire made more relevant for child completion | Increased ease of use for completion and relevance to stakeholder | Information sheet for child panel  Quarterly PIP newsbrief |
|  | Interview topic guides, questionnaire, consent form and study information sheets | 7^th^ – 15^th^ June 2021 | PIP (4) | Email |  | That parents were taking part in sub-study was made clearer.  Estimated time for length of interview was added. | Interview topic guide and questionnaire fit for purpose | Quarterly PIP newsbrief |
|  |  | 26^th^ June 2021 | Expert panel (3) | Email |  | Clarification that the interview and questionnaire is concerned with the NCMP **results** letters for parents.  ‘Provider service’ added to delivery teams options.  Additional questions added re changes in service provision and complaints and/or concerns from parents |  |  |
| **Study dissemination** | Blog about study PPI | 19^th^ April 2021 | PIP (1) | Email | We hoped for more feedback but did not have time to ask for more feedback due to blog deadline | Blog post confirmed as accurate and appropriate | Increased public and peer awareness of PPI methods and study | Quarterly PIP newsbrief |
|  | PPI section of funder’s annual report | 5^th^ July | PIP (2) | Email |  | Parents’ personal accounts of being a PIP member and impact of participation included in PPI section of report | Positive feedback received from funders – highlights the importance of PPI input | Quarterly PIP newsbrief |
| **Assessment of PIP** |  | 14^th^ December 2020 – 8^th^ February 2021 | PIP (7) | Online Survey |  | Make tasks quick and easy to complete (parents are busy people).  Ensure study demonstrates that input from PIP adds value. | PIP tasks checked for ease of understanding and completion by 2 research team members.  Regular newsletters and communication ensure parents are informed of PIP impact on study |  |
| **Study Website** | Preference for video or animation for additional health information videos | 16^th^ – 28^th^ June 2021 | PIP (5) | Email |  | Animation used for NCMP process information.  ‘Live’ videos used for information of conversation between health practitioner and parent and parent and child. | Additional health information is presented in a clear and acceptable format by parents | Quarterly PIP newsbrief |
|  | National Child Measurement Programme process information animation | 28^th^ August | PIP (4) | Email |  |  |  | Quarterly PIP newsbrief |
|  | Conversation between health professional and parent and parent and child information video scripts | 7^th^ – 12^th^ October 2021 | PIP (3) | Email |  | Practitioner-parent video: assumptions about family’s personal lifestyle lessened.  ‘Angry’ parent’s response changed to more appropriate response.  Practical lifestyle advice provided by practitioner.  Parent-child video: focus more on child being measured than on weight status. | Additional health information is presented in a clear and acceptable format by parents | Quarterly PIP newsbrief |
|  | Website testing | 29^th^ September 2021 | PIP (3) | Email |  | Minor technical glitches resolved.  Paragraph on the consequences of chid being above healthy weight revised to remove perception of ‘victim-blaming’.  ‘About MapMe2’ section amended to include more information about development of body image scales.  Differentiation between MapMe study and MapMe2 study made clearer. | Experience of, and journey through website made clearer and easier.  Accompanying text acceptable to parents. | Quarterly PIP newsbrief |
|  | Review of practitioner -parent and parent- child conversation video | 7^th^ October 2021 | PIP (3) | Email |  | Video divided into two separate films.  Video titles revised. | Parent can choose whether to watch one or both videos.  Made clearer what the videos contain. | Quarterly PIP newsbrief |
| **PIP Newsletters** |  | 22^nd^ October 2020  14^th^ December 2020  4^th^ March 2021  9^th^ June 2021  22^nd^ September 2021 | PIP | Email |  | Quarterly ‘newsbriefs’ emailed to parents who requested regular communication about the study progress and PIP input | Parent request for PIP input and impact acknowledged and fulfilled.  Ensures regular correspondence with group and encouragement to remain a member. |  |
| **Assessment of Child involvement** |  | October – November 2021 | Child panel (4) | Online survey |  | Clarification that 10-11 year olds enjoyed helping with study.  Use social media/ schools to get other children involved. | Future tasks made more accessible |  |
| **Trial Steering Committee meetings** |  | 30^th^ June 2021  8^th^ October 2021 | PIP (1)  PIP (2) | Zoom  Zoom | Following the first meeting, it was discovered that the PIP member was trying to join by Zoom and read online documents on a smart phone at the same time | Provide parents with meeting documents before meeting to aid those with limited screen access (mobile phone) | Meeting attendance made more accessible. |  |
| **Stakeholder and study team assessment of study PPI** | A values based card task based on PiiAF ^33^ | September – October 2021 | Stakeholders and Study team (3) | Padlet interactive platform | Response was lower than hoped | To consider other options of feedback/ interaction with busy professionals in future (multiple methods?) |  | Project meeting |
